# Pathological Characterization of Tumor Immune Microenvironment (TIME) in Malignant Pleural Mesothelioma

**DOI:** 10.3390/cancers13112564

**Published:** 2021-05-24

**Authors:** Francesca Napoli, Angela Listì, Vanessa Zambelli, Gianluca Witel, Paolo Bironzo, Mauro Papotti, Marco Volante, Giorgio Scagliotti, Luisella Righi

**Affiliations:** 1 Department of Oncology, University of Turin, 10043 Orbassano, Italy; francesca.napoli@unito.it (F.N.); vanessa.zambelli@unito.it (V.Z.); paolo.bironzo@unito.it (P.B.); mauro.papotti@unito.it (M.P.); marco.volante@unito.it (M.V.); giorgio.scagliotti@unito.it (G.S.); 2 Thoracic Oncology Unit, San Luigi Hospital, 10043 Orbassano, Italy; alisti@live.it; 3 Department of Medical Sciences, University of Turin, City of Health and Science, 10126 Torino, Italy; gianlucarobert.witel@unito.it; 4 Pathology Unit, City of Health and Science, 10126 Torino, Italy

**Keywords:** mesothelioma, tumor microenvironment, tumor-associated macrophages, dendritic cells, immunohistochemistry

## Abstract

**Simple Summary:**

Tumor immune microenvironment is an important structural component of malignant pleural mesothelioma that contributes to disease growth support and progression. Its study and pathological characterization are important tools to find new biomarkers for advanced therapeutic strategies.

**Abstract:**

Malignant pleural mesothelioma (MPM) is a rare and highly aggressive disease that arises from pleural mesothelial cells, characterized by a median survival of approximately 13–15 months after diagnosis. The primary cause of this disease is asbestos exposure and the main issues associated with it are late diagnosis and lack of effective therapies. Asbestos-induced cellular damage is associated with the generation of an inflammatory microenvironment that influences and supports tumor growth, possibly in association with patients’ genetic predisposition and tumor genomic profile. The chronic inflammatory response to asbestos fibers leads to a unique tumor immune microenvironment (TIME) composed of a heterogeneous mixture of stromal, endothelial, and immune cells, and relative composition and interaction among them is suggested to bear prognostic and therapeutic implications. TIME in MPM is known to be constituted by immunosuppressive cells, such as type 2 tumor-associated macrophages and T regulatory lymphocytes, plus the expression of several immunosuppressive factors, such as tumor-associated PD-L1. Several studies in recent years have contributed to achieve a greater understanding of the pathogenetic mechanisms in tumor development and pathobiology of TIME, that opens the way to new therapeutic strategies. The study of TIME is fundamental in identifying appropriate prognostic and predictive tissue biomarkers. In the present review, we summarize the current knowledge about the pathological characterization of TIME in MPM.

## 1. Introduction

Malignant pleural mesothelioma (MPM) is a rare and highly aggressive disease arising from pleural mesothelial cells. The recognized risk factors of MPM are asbestos, radiation exposure, genetic mutations, and the exposition to Simian Virus 40, but asbestos is certainly the most relevant and most well-known cause [1]. The overall prognosis of advanced stage MPM is poor, with a median survival of less than 15 months [2]. MPM consists of three histological variants: epithelioid (~60% of mesotheliomas), sarcomatoid, characterized by spindle cell morphology (~20% of mesotheliomas), and biphasic, which presents both epithelioid and sarcomatoid features (~20% of mesotheliomas) [3]. Diagnosis of MPM relies on an integration of clinical, radiological, and pathological findings, with histological examination being the mainstay for diagnosis and prognostication [4,5]. Since MPM is diagnosed in advanced stage in the majority of cases, the standard of care consists in systemic chemotherapy. However, the standard combination of cisplatin and pemetrexed chemotherapy agents [6] prolongs the median survival time by approximately 3 months only [7]. In the last years, genetic studies on MPM reported a low prevalence of oncogene driver mutations and low tumor mutational burden, but frequent copy-number losses and recurrent somatic mutations in oncosuppressor genes such as BAP1, NF2, and CDKN2A [8,9,10,11,12,13]. Unfortunately, no targeted therapies exploiting these alterations have emerged.

The etiopathogenetic evolution of MPM is mostly due to the generation of a tumor immune microenvironment (TIME) as a consequence of asbestos-induced damage, that may support tumor growth, possibly in association to genetic predisposition [14,15]. Over time, chronic inflammation determines an increased production of free radicals and reactive oxygen species by inflammatory cells and/or an alteration of immunocompetent cells, resulting in a reduction of tumor immunity [16].

The unique role of TIME in MPM development and progression still needs an accurate characterization in terms of infiltrating cell types, expression of co-inhibitory molecules, and activation of immune pathways (e.g., INFγ). As histological examination remains the gold standard in the diagnosis of MPM, the characterization of TIME could be crucial to visualize all cellular components and achieve a better understanding of the disease. Despite the different biological and clinical features between pleural and peritoneal mesothelioma [17], the presence of tertiary lymphoid structures (TLS) as a component of the host immune response was highlighted in epithelioid peritoneal mesothelioma (EMPM), as well. However, no association between TLS-EMPM and different oncological outcomes was found, thus suggesting that TLS would reflect an indirect mechanism of therapy resistance to drugs in EMPM as in its pleural counterpart [18].

Given the role of TIME in MPM, the use of immune checkpoint inhibition treatment has the rationale to provide new potential therapeutic opportunities. Indeed, the combination of monoclonal antibodies directed against programmed cell death protein 1 (PD-1) and cytotoxic T-lymphocyte-associated protein 4 (CTLA-4) recently showed its superiority over platinum-pemetrexed chemotherapy in a phase 3 trial [19]. Notably, a greater benefit was observed in biphasic/sarcomatoid MPM. Moreover, single-agent anti-PD-1 therapy demonstrated to significantly increase survival as compared to best supportive care in platinum pre-treated patients [20].

Another novel potential treatment in MPM is cell therapy. Clinical trials using CAR-T cells in MPM have shown that this potential therapy is relatively safe, but efficacy remains modest, likely due to the strong immunosuppressive conditions in MPM microenvironment [21,22]. Furthermore, preclinical studies are ongoing in a bimodal treatment approach consisting of dendritic cell (DC) vaccination to prime tumor-specific T cells, a strategy to reprogram the desmoplastic microenvironment in mesothelioma and pancreatic cancer [23].

**Figure 1:**
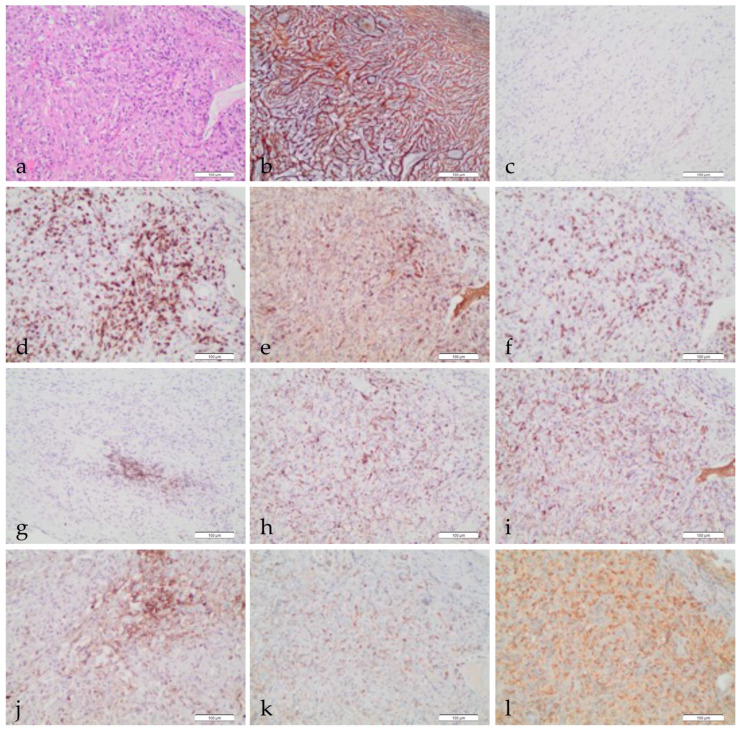
Pathological characterization of TIME in MPM. Histological appearance of MPM, epithelioid type (**a**), Ematoxilin & eosin (100×); reticulin stain showing connective tissue around neoplastic cells (**b**), (100×); SMA IHC stain showing scattered fibrocytes (**c**), (100×); CD3 IHC stain highlighting T lymphocytes (**d**), (100×); CD4 IHC stain showing scattered T cells (**e**), (100×); CD8 IHC stain showing moderate T lymphocyte infiltrate (**f**), (100×); CD20 IHC stain showing a small aggregate of B lymphocytes (**g**), (100×); CD68 IHC stain showing diffuse macrophage infiltration (**h**), (100×); CD163 IHC stain showing activated TAMs (**i**), (100×); PD-L1 IHC stain showing small aggregates of positive tumor cells (**j**), (100×); VISTA IHC stain showing moderate expression in immune cells (**k**), (100×); STING IHC stain showing diffuse immune cell positivity (**l**), (100×). Notes: TIME: tumor immune microenvironment; MPM: malignant pleural mesothelioma; SMA: smooth muscle actin; IHC: immunohistochemistry; PD-L1: programmed death ligand 1; VISTA: V-domain Ig-containing suppressor of T-cell activation; STING: STimulator of Interferon Genes.

The most adequate tissue specimens for MPM pathological characterization derive from video-assisted thoracoscopic (VATS) biopsies or pleurectomies, which are the recommended samples for complete histological diagnosis [2,3,7]. The availability of large amounts of tissue allows both the definition of histological tumor features and immune cells’ spatial distribution on routine hematoxylin-eosin slide. On these specimens, the cheapest and fastest tool used for pathological characterization studies is immunohistochemistry (IHC), that allows to visualize both tumor cells and microenvironment components, according to their immunophenotype and biomarker expression (Figure 1). Despite its advantages, a limitation of chromogen-based IHC analysis is the impossibility of using more than one or two markers per slide. Novel and innovative multiplex immunophenotyping techniques are in development to deeply analyze as a whole both the spatial distribution and immunophenotypic interaction of each single cell subtype [24,25,26].

Given the need to explore TIME in its components and constituents, in this review, we summarize the current data on TIME pathological characterization and biomarker identification in MPM.

## 2. The Tumor Immune Microenvironment

TIME is a complex and heterogeneous mixture of stromal, endothelial, and immune cells admixed in a connective matrix and its composition differs among individuals and histological types. In fact, studies suggest that TIME profoundly differs between epithelioid and non-epithelioid pleural mesotheliomas: the former typically have an immune-activated TIME with greater proportion of plasmacytoid dendritic cells (DC), CD20+ B cells, CD4+ helper T cells, and exhausted CD8+ tumor-infiltrating lymphocytes (TILs), whereas non-epithelioid mesotheliomas have a TIME with a larger proportion of macrophages, regulatory T cells, mesothelioma stem cells, and neutrophils [27].

In past years, the prognostic and predictive role of TIME in MPM was investigated mainly on small and heterogeneous series, with no conclusive data due to difficulties in MPM microenvironment characterization [28,29]. Moreover, qualitative and quantitative changes in tumor/stroma ratio may produce a dramatic rewiring in the MPM-infiltrating immune cell subsets [30].

Increasing evidence suggests that analysis of gene expression or copy numbers in cancer samples helps to understand immune cell infiltration into the tumor ME. Yoshihara et al., by means of transcription profiling, have developed the ESTIMATE algorithm (Estimation of STromal and Immune cells in MAlignant Tumor tissues using Expression data) to analyze the stromal and immune infiltration associated to tumor cellularity in cancer samples [31]. Using gene expression data, a ‘stromal signature’, that describes the presence of stroma in tumor tissue, and an ‘immune signature’, that represents the infiltration of immune cells, were identified. Recently, the ESTIMATE algorithm was applied to MPM samples and the involvement of 14 immune/stromal-related genes was found to have significant prognostic potential. In silico analyses revealed that all these genes are involved in immune responses and may predict the survival of patients with MPM, playing also a role as biomarkers of the sensitivity to immunotherapy [32].

Additionally, Lee and coworkers, using mass spectrometry and comprehensive analysis of intra-tumoral immune system, described a distinct immunogenic TIME signature which was associated with favorable OS and response to checkpoint blockade [33]. The importance of understanding TIME of different MPM histotypes in relation to hypo-fractionated radiation therapy response was recently demonstrated as well [27].

### 2.1. Extracellular Matrix and Stroma Components

In MPM, the intra-tumoral stroma is not merely a scaffold but also promotes tumor growth, invasion, and protection from an anti-tumor immune response.

Several studies reported that many genes involved in extracellular matrix (ECM) production and remodeling are upregulated in MPM, especially in the biphasic [34] and sarcomatoid [35] variants. Furthermore, increased expression of these ECM-related genes is associated with “immune desert” tumor regions, characterized by a poor lymphocytic infiltrate, suggesting that MPM-altered stroma might act as a barrier to the immune response [36]. Very recent studies that analyze public mRNA-sequencing datasets through bioinformatic analyses have identified several differentially expressed genes (DEGs) in MPM. In these studies, genes specifically associated to the ECM component, structural constituents, organization, and receptor interaction were found overexpressed. These genes resulted in being involved in different protein–protein interaction (PPI) networks, gene ontology (GO), biological processes (BC), and molecular functions [37,38], and were also validated in MPM cell line models [39].

ECM components such as collagen, laminin, fibronectin, and integrins can be produced by mesothelioma cells that can also promote, under the influence of various growth factors, the synthesis of matrix metalloproteases (MMP), favoring ECM remodeling and tumor cell invasion [40].

In vitro studies demonstrated that different histotypes are characterized by specific ECM profiles, and that these differences determine a varying ability of MPM cells to spread and migrate towards ECM substrates [41,42]. In particular, characterization of cell culture conditions showed that 3D growth of malignant cells was enhanced in the presence of their own ECM, while invasion was stimulated by fibronectin in epithelioid and biphasic MPM histotypes, while homologous cell-derived ECM stimulated invasion in the most aggressive (sarcomatoid) form of MPM.

Furthermore, inhibition of collagen production delays MPM tumor growth [43]. Morphometric and immunohistochemical analysis of tumor collagen V (Col V), along with the quantitative inverse relationship between Col V and CD8+ T lymphocytes, demonstrated that high levels of Col V and low CD8+ T lymphocytes confer an immune-privileged TIME for tumor invasion and poor patients’ prognosis [44,45].

The architecture of connective tissue in MPM per se, highlighted by silver-based reticulin staining (Figure 1b), has been recently proposed to distinguish the transitional variant of MPM, showing intermediate features between epithelioid and sarcomatoid histotypes, and bearing a specific prognosis [46]; in fact, a delicate reticulin pattern around single cells is indicative of this transitional type, as compared to a rough pattern banding individual cells in the sarcomatoid, and a large cluster pattern in the epithelioid type [47].

#### Cancer-Associated Fibroblasts (CAFs)

Tumor stroma is mostly composed by both fibrocytes with small spindle-shaped nuclei, derived from macrophages or dendritic cells, and activated fibroblasts (or cancer-associated fibroblasts, CAFs) that are identified by alpha smooth muscle actin (SMA) (Figure 1c) [48,49].

In recent years, fibroblast growth factor receptor (FGFR) signaling has been recognized as increasingly important, both in cancer pathogenesis and as a potential therapeutic target [50]. There are strong preclinical data suggesting that FGF is important in MPM as well. In MPM cell lines, FGFR1 and FGFR2 are co-expressed and their expression is strongly associated with sensitivity to FGFR-active tyrosine kinase inhibitors [51]. Inhibiting FGF autocrine signaling using an FGF-ligand trap reduces proliferation in MPM cell lines and reduces tumor growth in xenografts [52]. Unfortunately, the phase II clinical trial with a FGFR 1–3 inhibitor did not demonstrate efficacy in patients with MPM, who had progressed after first-line treatment with platinum-based chemotherapy [53].

CAFs have been shown to exert pro-tumorigenic effects by secreting several growth factors that promote cancer cell proliferation and invasion [54]. Literature data reported that TGFβ, IL-6, and CCL2, synthetized by CAFs [55], were detected in pleural effusions of MPM patients, where they seem to contribute to the recruitment and differentiation of immunosuppressive cells [56,57].

Our group identified Caveolin 1 (CAV1)-positive CAFs in a subgroup of epithelioid MPM with poorer prognosis [58]. CAV1 acts as a multifunctional scaffolding protein with multiple binding partners and is associated with cell surface caveolae in the regulation of lipid raft domains, but it is also involved in cancer growth and progression, modulating tissue responses through architectural regulation of the microenvironment. Recently, caveolae and their components emerged as integrators of different cell functions, mechano-transduction, and ECM–cell interactions [59]. Furthermore, in vitro studies on quantitative proteomic profiling revealed that CAV1 is required for exosomal sorting of ECM protein cargo subsets and for fibroblast-derived exosomes to efficiently deposit ECM and promote tumor invasion of breast cancer cells [60].

Furthermore, connective tissue growth factor (CTGF), a pro-tumorigenic CAF marker [49], is more expressed in sarcomatoid than in epithelioid MPM [61], and it is produced by both MPM cells and fibroblasts, and promotes the invasion of MPM cells in vitro [62]. Ohara’s group has demonstrated that a CTGF-specific monoclonal antibody (FG-3019, pamrevlumab) could inhibit mesothelioma cell growth in vitro [63]. Based on these data, it was suggested that the use of FG-3019, currently under clinical trials for idiopathic pulmonary fibrosis [64] and pancreatic ductal adenocarcinoma [65], could be a therapeutic option for MPM. This is supported by preclinical data including a strong in vivo cancer growth inhibition observed in melanoma and pancreatic cancer with the use of the same anti-CTGF monoclonal antibody [66,67].

### 2.2. Inflammatory Cellular Component of TIME

#### 2.2.1. Tumor-Associated Macrophages

Macrophages are specialized phagocytic cells that play a dual role in cancer depending on their differentiation. Tumor-associated macrophages (TAMs) derive from circulating monocytic precursors and are the major component of MPM TIME (Figure 1h,i). They are divided into two classes: classically activated (M1) macrophages, which have pro-inflammatory, tissue destructive, and anti-tumor activity, and alternatively activated (M2) macrophages, which have pro-tumorigenic properties [68]. M2 macrophages are the ones mostly present in MPM and their differentiation is regulated by interleukins, such as IL-4, IL-13, and IL-10, produced by tumor-infiltrating lymphocytes (TILs) [69].

Asbestos phagocytosis by macrophages triggers the formation of the inflammasome complex and promotes secretion of IL-1β [70,71]. Additionally, IL-1β/IL-1 receptor (IL-1R) signaling was reported to contribute to the oncogenesis of asbestos-induced mesothelioma [72]. These studies highlight the important role of the inflammasome in MPM development. The phagocytosed asbestos fibers remain undegraded and induce apoptosis of macrophages [73]. Undegraded asbestos fibers then undergo phagocytosis by nearby macrophages. Thus, asbestos is not completely removed and constitutively activates the inflammasome in macrophages. Moreover, it was reported that high mobility group box 1 (HMGB1) protein is abundantly secreted by MPM cells and serum levels of HMGB1 are associated with poor prognosis in MPM patients [74,75]. HMGB1 is one of the damage-associated molecular pattern proteins and promotes pro-IL-1β production functioning as an agonist of Toll-like receptor 4 (TLR4) [76]. Both HMGB1 derived from MPM cells and asbestos-activated inflammasome in TAMs induce IL-1β production, resulting in enhanced aggressiveness of MPM [77].

The tissue localization of M2 macrophages has been investigated in different immunohistochemical studies. Marcq and coworkers demonstrated that the number of stromal CD68+ macrophages found in MPM specimens was positively correlated to the number of stromal Tregs, suggesting a direct action of macrophages on stimulating and recruiting CD4+ immunosuppressive cells [78]. Burt et al. found that the absolute number of CD68+ macrophages was associated with worse prognosis in non-epithelioid MPM [79]. Finally, Cornelissen and coworkers reported that patients who develop recurrence after radiation treatment have a higher M2/total TAM ratio and lower CD8+ cell count at diagnosis, compared to patients who did not develop this outgrowth [80].

#### 2.2.2. T Cells and Natural Killer Cells

The CD3+ T-lymphocytes are the second most common immune cell type in MPM (Figure 1d–f) TIME and constitute, on average, 20–42% of the immune cell infiltrate [68,81]. T helper CD4+ cells play an important role in the generation of T cell-mediated antitumor response via activation of antigen-presenting cells (APCs), which stimulate CD8+ cytotoxic TILs and natural killer (NK) cells. The latter are lymphoid cells of the innate immune system with strong immunostimulatory functions and cytotoxic capacity [82].

A recent study by Alay and coworkers, performing an integrative transcriptome analysis on a publicly available dataset of 516 MPMs, revealed a clinically relevant immune-based classification based on CD4+ T-helper 2 (TH2) and CD8+ cytotoxic T cells, that were found to be consistently associated with better overall survival [83].

CD8+, CD4+, and CD4+/FoxP3+ T-cells are present in the majority of patients [84], but the number of T-reg cells in pleural effusions of MPM patients is lower than in other solid tumors [85], confirming the presence of an immunosuppressive milieu in MPM tumoral mass, rather than in pleural effusion [86]. The positive effect of CD4+ tumor-infiltrating lymphocytes (TILs) on prognosis has been previously suggested for epithelioid [78,87,88,89], but remains controversial in sarcomatoid MPM [81,88]. On the other hand, low CD8+ and high FoxP3+ TILs counts were shown to correlate with a high risk of both death and recurrence, regardless of the presence of a sarcomatoid component [87,88,89,90].

Ujiie and coworkers demonstrated the prognostic role of CD8+ and CD20+ expressing lymphocytes in 230 epithelioid mesothelioma patients [29]. In particular, they found that rather than the single type of infiltrating cells, the combination of high M2-polarized TAMs (CD163^+^) with low CD8+ T cells, and low M2-polarized TAMs (CD163^+^) with high CD20+ B cells, were independent markers of worse and better overall survival, respectively. These data were confirmed by Pasello et al., except for the fact that CD8+ T-lymphocytes were found in MPM samples showing aggressive features (sarcomatoid/biphasic histology, higher necrosis, and proliferation index), when associated with higher CD68+ macrophages and PD-L1 expression [90].

In a study by our group, Salaroglio et al. [91] performed a simultaneous comprehensive analysis of the immune infiltrate in pleural fluid and fresh pleural biopsy tissues aiming to identify an immune phenotype with diagnostic and prognostic value in MPM patients. It was confirmed that CD8+ TILs in pleural effusion have no prognostic significance, while intratumor immune infiltrate is more effective in predicting the patient’s outcome. The same result was obtained by Chee et al., who state that high proportions of FoxP3+ T cells are associated with a poor prognosis in epithelioid and sarcomatoid tumors [88].

Moreover, Fusco et al. found an increased presence of stromal CD4+ T and CD19+ B lymphocytes with a positive correlation between each other, possibly indicating a positive feedback loop between these two lineages [92].

Our group also characterized TIME in MPM by immunohistochemistry, as a validation step of gene expression profiling. In MPM cases with higher expression of T-cell lineage genes, T-effector genes, and T-regulatory genes, we observed a high expression of CD3+ T-infiltrating lymphocytes, with a similar amount of CD4+ and CD8+ T-cells. On the contrary, high amounts of CD20+ B lymphocytes, with follicular chronic inflammation as a morphological hallmark, were observed in the group that showed higher relative expression of B cell and lower expression of T cell genes [36].

#### 2.2.3. Myeloid-Derived Suppressor Cells

Myeloid-derived suppressor cells (MDSC) are myeloid cells with suppressive activity on innate and adaptive immune cells that have been described to inactivate immune response against the tumor in cancer patients [93]. Based on their surface markers, MDSC can be subdivided in granulocytic MDSC (GR-MDSC), which express granulocytic markers like CD66b and/or CD15, and monocytic MDSC (MO-MDSC), which express the monocytic antigen CD14 [91]. The main mechanisms by which MDSC exert their suppressive activity on other immune cells are the depletion of arginine and tryptophan by expression of effector enzymes arginase I (Arg I), inducible NO-synthase (iNOS), and indolamin-2,3-dioxygenase (IDO), as well as by production of reactive oxygen species (ROS) [93].

In mice, MDSCs are characterized by IL-4 expression [94]. Burt et al. found IL-4R to be highly expressed on the surface of human MPM tumor cells: IL-4R was present in 97% of epithelial and 95% of non-epithelial tumors. Only a scattered and small fraction of stromal cells stained positive for IL-4R, and conversely, IL-4R-positive macrophages were predominantly found in the stroma [95]. Myeloid CD33+ cells were found to represent approximately 42% of CD45+ immune cells: 0.6–31% of these myeloid cells were typed as MDSCs [96].

In their study, Salaroglio et al. reported that GR- and MO-MDSCs abrogated proliferation and cytotoxic activity of autologous TILs and of TILs derived from patients with pleuritis, suggesting an important role of MDSCs in immunosuppression mediation. Moreover, the intratumor-infiltrating MDSCs, but not the MDSCs of pleural fluid, resulted significantly associated with poorer PFS and OS [91].

Furthermore, it was recently reported that MPM TIME is enriched in infiltrating granulocytes, which inhibit T-cell proliferation and activation. Immunohistochemistry and transcriptomic analysis revealed that a majority of MPMs express GM-CSF, and that high GM-CSF expression correlates with clinical progression. Blockade of GM-CSF with neutralizing antibodies or ROS inhibition restores T-cell proliferation, suggesting that targeting GM-CSF could be of therapeutic benefit in MPM patients [97].

#### 2.2.4. Dendritic Cells

Dendritic cells (DCs) are powerful antigen-presenting cells with key roles in the initiation and regulation of immune responses. DCs are unique in their ability to activate naïve T cells and initiate primary immune responses in lymph nodes, and they also play a central role in reactivating memory T-cell responses in the lungs. DC-derived signals regulate both the degree of T-cell activation and the nature of immune response (e.g., T helper (Th) 1, Th2, Th17, B-cell help) [98]. Several DC subpopulations have been defined: DCs are broadly divided into myeloid dendritic cells (mDCs), usually referred to as conventional dendritic cells (cDCs), and plasmacytoid dendritic cells (pDCs). In human lungs, cDCs form dense networks throughout the epithelium of large conducting airways, bronchioles, alveoli, and interstitial space, and they express CD141, CD1c, and the C-type lectin domain family 9 member A (CLEC9A) [99,100]. pDCs are best characterized by their ability to synthesize great amounts of IFN. They are relatively inefficient at presenting antigens to T cells and seem to play an important role in tolerance induction, probably via induction of regulatory T cells. In humans, pDCs are identified by surface markers such as CD303 (a C-type lectin), CD304 or neuropilin-1, Ig-like transcript 7, and IL-3 receptor-a chain [101]. Under normal conditions, activated pDCs exhibit robust IFN-α production and promote both innate and adaptive immune responses. In several cancer models [102], including MPM [103], pDCs demonstrate an impaired response to T activation, decreased or absent IFN-α production, and contribute in establishing an immunosuppressive TIME and a reduced ability to generate effective anti-mesothelioma T cell responses. On the other hand, a comprehensive proteomic analysis on 12 surgically resected MPMs highlighted a correlation between the presence of activated pDCs (CD40+ and CD86+) and tumors having a good TIME signature as well as a favorable response to immune checkpoint therapy [33]. Finally, evidence to date suggests that CD40+ DC activation is a critical and nonredundant mechanism to convert “cold” tumors (i.e., lacking a T cell tumor infiltrate) into “hot” ones (i.e., having a prominent T cell tumor infiltrate), sensitizing them to checkpoint inhibition therapy [104,105].

#### 2.2.5. B Lymphocytes

B lymphocytes contribute to humoral immunity as they can differentiate into antibody-secreting plasma cells. Additionally, B cells can stimulate T cells or serve as APCs. In MPM, B lymphocyte infiltrate is associated with better patient survival [29,90]. Generally, B cell infiltrate in mesothelioma is scant [55,89].

As mentioned above, in our study on immune gene expression profiling in MPM, the subgroup with downregulated T-cell effector and upregulated B-cell genes failed to show correlation with increased expression of genes associated with antigen presentation, thus we concluded that these B cells may be part of the adaptive cytotoxic response [36].

### 2.3. PD-L1 and Other Immune Checkpoints

The programmed cell death pathway (PD-1/PD-L1) plays a critical role in tumor immune escape control. PD-1 is mainly expressed on activated CD4/CD8 T cells and B cells [96]. PD-L1, the ligand of PD-1, is not only expressed in immune cells, but also in others, including cancer cells, helping immune evasion by interacting with PD-1 on T-cells [106]. The interaction between tumor PD-L1 and PD-1 on T cells results in the inhibition of T cell activation and proliferation, as well as immune evasion by PD-L1-expressing tumors [107].

PD-L1 immunohistochemical expression in tumor tissue has been widely accepted as a predictive biomarker [108], because of its association with increased efficacy of immune checkpoint inhibitors (ICIs) in several malignancies [109]. Immunotherapy based on monoclonal antibodies against PD-1 and PD-L1 has also been tested for MPMs in clinical trials (Figure 1j). Several nonrandomized phase I/II trials, testing single-agent ICI, showed variable antitumor activity (9–29%) and median progression-free survival ranging from 2.8 to 6.2 months [110]. Preliminary results from phase II clinical trials combining inhibitors of cytotoxic T-lymphocyte-associated antigen 4 (CTLA4) and anti-PD1/PD-L1, such as ipilimumab, nivolumab, tremelimumab, or durvalumab, showed promising results but significant toxicity [111]. In those clinical trials, PD-L1 expression showed limited value in predicting benefit from ICIs, and PD-L1 expression analysis currently has no role as a clinical predictive biomarker in MPM. Moreover, the prognostic value of PD-L1 expression in MPM is controversial. In a recent meta-analysis, Jin et al. reported that PD-L1 overexpression significantly correlated with poor overall survival, irrespective of the sample size of the series, treatment method, or PD-L1 cut-off value. Furthermore, overexpression of PD-L1 was associated with sarcomatoid and biphasic histology [112].

The above-mentioned integrative transcriptome analysis of MPM [83] revealed a clinically relevant immune-based classification of the same, identifying three immune groups (IG1–IG3) that represent different immune infiltration patterns and are associated with distinct survival outcomes. The group with the shortest overall survival (IG1) represented more than 50% of cases, whereas the IG3 group, having the best prognosis, accounted for 8.5% of cases only. Interestingly, while most immune checkpoint markers correlated with the different immune groups, CD276 (B7-H3) showed an opposite expression pattern, decreasing from IG1 to IG3. CD276 is a member of the B7 family of immunoregulatory proteins and is overexpressed in several tumor types. It has been shown that CD276 can promote tumor proliferation, angiogenesis, and metastasis, and is associated with shorter survival time in multiple tumor types [113]. A recent study reported a wide immunohistochemical expression of B7-H3 in MPM and demonstrated that PD-L1 and B7-H3 were significantly co-expressed in tumor cells of the non-epithelioid histotype [114]. Similarly, CD44 is the only T-cell exhaustion marker that showed negative correlation with the immune groups [83]. This marker has been associated with metastasis and low survival rates in multiple cancer types [115]. In MPM, CD44 has been shown to promote invasiveness when interacting with hyaluronan [116,117].

V-domain Ig-containing suppressor of T-cell activation (VISTA) is another immune checkpoint that inhibits anti-tumor immune responses (Figure 1k). In a TCGA-based study, VISTA gene expression was reported to be higher in MPM than in all other cancer types. This was particularly observed in the epithelioid subtype and strongly correlated with mesothelin expression [11]. Moreover, VISTA was recently described as a new potential target for mesothelioma immunotherapy. Muller et al. investigated the tissue expression of VISTA and PD-L1 in a large cohort of MPMs. They found frequent expression of VISTA and infrequent expression of PD-L1 (88% and 33% of epithelioid, 90% and 43% of biphasic, and 42% and 75% of sarcomatoid) with favorable and unfavorable survival correlations, respectively [118].

In this context, the expression of STimulator of Interferon Genes (STING) protein is described as having a crucial role in identifying “inflamed” or “hot” tumors that could be successfully treated with immunotherapy (Figure 1l). STING absence implies a tumor environment with no activation of the INFγ pathway, which is a known parameter of response to ICIs [119]. Moreover, it has been reported that targeting DNA damage response promotes anti-tumor immunity through STING-mediated T-cell activation in small-cell lung cancer [120].

## 3. Angiogenesis

The prognosis of MPM is best explained by a continuous model, which shows specific expression patterns of genes involved in angiogenesis and immune response [121]. Asbestos fibers have a direct effect on mesothelial cells, causing the release, together with inflammatory cytokines, of vascular endothelial growth factor (VEGF), which attracts leukocytes [122]. VEGF signaling is crucial in MPM pathophysiology [123], regulating blood vessel function, inducing tumor cell growth, and suppressing immune activation [124]. VEGF also acts as a powerful mitogen for mesothelial cells themselves. Indeed, MPM cell lines secrete VEGF-A and VEGF-C, as well as expressing both VEGF receptors Flt-1 (VEGF-R1) and KDR (VEGFR-2) [125,126]. Thus, VEGF signaling can induce MPM cell growth in an autocrine fashion. This may explain why mesothelioma cells show striking sensitivity to anti-VEGF agents, in addition to the more canonical role of such agents in inhibiting neo-angiogenesis. Moreover, MPM has been shown to produce the highest levels of VEGF among solid tumors [127].

Other growth factors can also regulate migration, survival, and differentiation of endothelial cells, contributing to neoangiogenesis, such as TGFb, EGF, angiogenin, IL-8, and platelet-derived growth factor (PDGF) [128]. All this evidence provides the rationale for the development of VEGF and angiogenesis inhibitors as a therapeutic strategy in MPM [129].

Although there has been over a decade of intense investigation, there are still no validated biomarkers of angiogenesis able to predict the efficacy of anti-angiogenic agents both in MPM and in other cancers [130]. The complementary LUME-Meso biomarker study has reviewed the plasma levels of 58 angiogenic factors and single-nucleotide polymorphisms (SNPs) in genes for VEGFR1 (FLT1), and VEGFR3 (FLT4) and mesothelin (MSLN), and assessed micro-vessel density via CD31 immunohistochemistry on archival biopsy samples. Although PFS and OS benefits were observed in patients with low plasma endoglin and homozygous VEGFR1/3 genotypes, no biomarkers showed any significant and conclusive association with antiangiogenic efficacy [131].

Recently, Chia and coworkers evaluated VEGF, PDGF, FGFR, and CD31 by immunohistochemistry in tissue microarrays from 329 patients who underwent surgical resection or biopsy for MPM. They found that high CD31 density and high PDGF expression levels were associated with poor prognosis in the epithelioid MPM group [132].

## 4. Conclusions

TIME is a challenging component with an emerging pathogenic, immunomodulatory, and growth-promoting role in MPM. Given the relatively low mutational burden of MPM, biological events other than genetics may be critical determinants of MPM growth and aggressiveness and could influence cells’ immune-escape.

**Figure 2:**
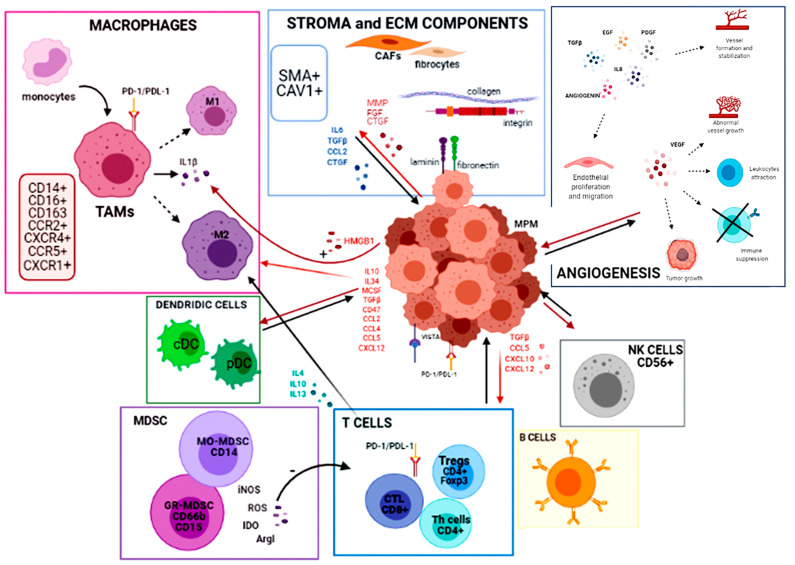
Graphical representation of tumor immune microenvironment interactions in MPM.

A greater understanding of infiltrating immune cells, their role and function, and the presence of ligand or modulatory marker expression will give a wider and better structured picture of the tumor–immune cell interplay (Figure 2).

A precise pathological and immuno-phenotypical characterization of TIME, in terms of extracellular matrix profiles, subtypes of immune-infiltrating cells, expression of co-inhibitory molecules, and activation of immune pathways could provide important knowledge for translational pathology studies. Practical identification of specific biomarkers that could influence the host immunity has to be performed and would represent a major advance for clinical translation of neoantigen-directed immunotherapies, paving the way to understand how to personalize future therapeutic approaches in MPM patients.
